# Computer-assisted planning for minimally invasive anterior two-thirds laser corpus callosotomy: A feasibility study with probabilistic tractography validation

**DOI:** 10.1016/j.nicl.2020.102174

**Published:** 2020-01-13

**Authors:** Vejay N. Vakharia, Rachel E. Sparks, Sjoerd B. Vos, Yarema Bezchlibnyk, Ashesh D. Mehta, Jon T. Willie, Chengyuan Wu, Ashwini Sharan, Sebastien Ourselin, John S. Duncan

**Affiliations:** a Department of Clinical and Experimental Epilepsy, University College London, London, UK; b Chalfont Centre for Epilepsy and National Hospital for Neurology and Neurosurgery, Queen Square, London, UK; c School of Biomedical Engineering and Imaging Sciences, King's College London, London, United Kingdom; d Centre for Medical Image Computing, University College London, London, United Kingdom; e Department of Neurosurgery and Brain Repair, University of South Florida, Tampa, Florida, United States; f Northwell Health Neuroscience Institute, New York, United States; g Department of Neurological Surgery, Emory University Hospital, Atlanta, Georgia, United States; h Division of Epilepsy and Neuromodulation Neurosurgery, Vickie and Jack Farber Institute for Neuroscience, Thomas Jefferson University, Philadelphia

**Keywords:** Computer-assisted planning, Laser interstitial thermal therapy, Corpus Callosotomy

## Abstract

**Background:**

Anterior two-thirds corpus callosotomy is an effective palliative neurosurgical procedure for drug-refractory epilepsy that is most commonly used to treat drop-attacks. Laser interstitial thermal therapy is a novel stereotactic ablative technique that has been utilised as a minimally invasive alternative to resective and disconnective open neurosurgery. Case series have reported success in performing laser anterior two-thirds corpus callosotomy. Computer-assisted planning algorithms may help to automate and optimise multi-trajectory planning for this procedure.

**Objective:**

To undertake a simulation-based feasibility study of computer-assisted corpus callostomy planning in comparison with expert manual plans in the same patients.

**Methods:**

Ten patients were selected from a prospectively maintained database. Patients had previously undergone diffusion-weighted imaging and digital subtraction angiography as part of routine SEEG care. Computer-assisted planning was performed using the EpiNav™ platform and compared to manually planned trajectories from two independent blinded experts. Estimated ablation cavities were used in conjunction with probabilistic tractography to simulate the expected extent of interhemispheric disconnection.

**Results:**

Computer-assisted planning resulted in significantly improved trajectory safety metrics (risk score and minimum distance to vasculature) compared to blinded external expert manual plans. Probabilistic tractography revealed residual interhemispheric connectivity in 1/10 cases following computer-assisted planning compared to 4/10 and 2/10 cases with manual planning.

**Conclusion:**

Computer-assisted planning successfully generates multi-trajectory plans capable of LITT anterior two-thirds corpus callosotomy. Computer-assisted planning may provide a means of standardising trajectory planning and serves as a potential new tool for optimising trajectories. A prospective validation study is now required to determine if this translates into improved patient outcomes.

## Highlights

• Laser interstitial thermal therapy (LITT) is a novel minimally invasive technique for the treatment of epilepsy.
• We test computer assisted planning with LITT to disrupt seizure spread.
• Trajectory parameters and models were automatically generated from a single T1 image.
• Probabilistic tractography revealed comparable interhemispheric disconnection to blinded expert surgeons.

## 1 Introduction

Open corpus callosotomy was first described by van Wagenen and Herren in 1940 to prevent “*the disordered wave of nerve impulses… spreading widely to other parts of the neopallial portion of the brain*” (VAN WAGENEN and HERREN, 1940). . The corpus callosum is the principal site of interhemispheric connectivity consisting of both myelinated and unmyelinated fibres. In modern epilepsy surgery, corpus callosotomy is performed as a palliative procedure for drug-refractory focal epilepsy (Vakharia et al., 2018a). Disconnection of interhemispheric connectivity through the anterior two-thirds of the corpus callosum is a highly effective palliative procedure that is most commonly undertaken for drop attacks and tonic, atonic or tonic-clonic seizures as part of Lennox-Gastaut syndrome. Pre-operative independent prognostic factors include younger age, drop attacks with an associated epilepsy syndrome, MRI negative and IQ > 50 (Asadi-Pooya et al., 2008). A less invasive and reversible alternative to corpus callosotomy is vagal nerve stimulation (VNS). A meta-analysis comparing corpus callosotomy with VNS found corpus callosotomy to be significantly more effective than VNS in reducing atonic seizure frequency in patients with Lennox-Gastaut syndrome (Lancman et al., 2013), but due to the less invasive nature VNS is more commonly undertaken first (Englot et al., 2017). Less common indications for corpus callosotomy include treatment for refractory recurrent status epilepticus, refractory complex partial seizures with the rapid secondary generalisation of presumed frontal lobe onset and no obvious focus, refractory generalized tonic-clonic and refractory absence seizures (Asadi-Pooya et al., 2008).

Minimally invasive approaches to anterior two-thirds corpus callosotomy have been described utilising stereotactic radiosurgery (Pendl et al., 1999) and laser interstitial thermal therapy (LITT) (Ho et al., 2016; Lehner et al., 2018; Palma et al., 2018; Pruitt et al., 2017). We aimed to develop a computer-assisted planning algorithm to optimise the interhemispheric disconnection and associated safety metrics for LITT anterior two-thirds corpus callosotomy. As part of a simulation-based feasibility study, we compare computer-assisted planning derived trajectories with blinded expert manual plans in 10 patients and quantify the extent of disconnection using fibre tractography. This is carried out to establish the feasibility of the approach in epilepsy patients without gross structural abnormalities.

## 2 Methods

### 2.1 Patient inclusion

LITT corpus callosotomy trajectory planning was performed on 10 patients (6 male) selected from a prospectively maintained database. As this is a simulation-based feasibility study, patients were selected on the basis of having a structurally normal MRI scan and being eligible for corpus callosotomy, but not necessarily having had this performed at the time of the study. Further selection criteria included having undergone bilateral digital subtraction angiography (DSA), as part of their routine stereoelectroencephalography (SEEG) care, and pre-operative diffusion-weighted imaging. All patients had previously undergone SEEG implantation at the National Hospital for Neurology and Neurosurgery between 2017 and 2019. Computer-assisted planning was undertaken prior to manual planning by two independent externally blinded experts (YB and JTW).

### 2.2 Ethical approval

Ethical approval for this study was provided by the National Research Ethics Service Committee London, approval reference: 12/LO/0377. Written consent was obtained from all patients prior to inclusion in the study.

### 2.3 Computer-assisted planning algorithm

#### 2.3.1 Image acquisition and model generation

**Fig. 1:**
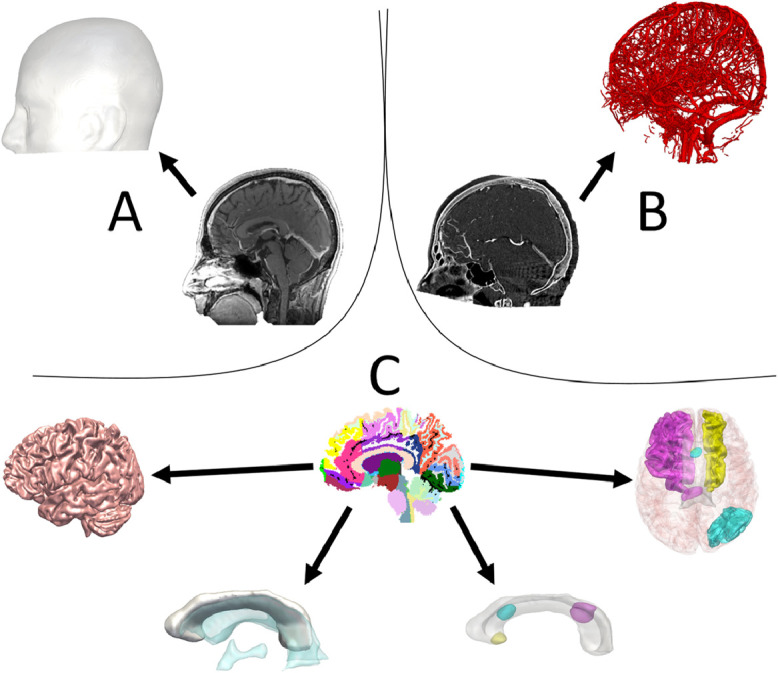
Legend: Model generation: A) Scalp model generated from the T1 image through thresholding. B) Vascular segmentation generated from raw digital subtraction angiography acquisition. C) Cortex, corpus callosum, ventricular system, anterior, middle and posterior target points and corresponding entry regions generated from GIF parcellation.

Image requirements for the automated pipeline included a 1 mm isotropic 3D-T1 magnetization-prepared rapid acquisition with gradient echo (MPRAGE) (TE/TR/TI 3.1/7.4/400 ms, field of view (FOV) 224 × 256 × 256 mm, matrix 224 × 256 × 256), gadolinium-enhanced T1 SPGR (T1+Gad) and digital subtraction angiography (DSA) images. A whole brain parcellation and pseudoCT image were generated from the T1 MPRAGE sequence using geodesic information flows (GIF) (Burgos et al., 2014; Cardoso et al., 2015). Models of the cortex, lateral ventricles, non-dominant superior frontal gyrus, dominant superior frontal gyrus, dominant middle frontal gyrus non-dominant superior parietal lobule and non-dominant angular gyrus were automatically segmented from the GIF parcellation (see Fig. 1 and Table 1).

**Table 1:** LITT Callosotomy default parameters:

Trajectory no.	Entry ROI	Target ROI	Max Length*	Max Angle
1	Right Superior Frontal Gyrus	Anterior Target	60	15
2	Right Superior Parietal Lobule	Middle Target	110	35
3	Right Angular Gyrus	Middle Target	110	35
4	Left Superior Frontal Gyrus	Posterior Target	90	35
5	Left Middle frontal Gyrus	Posterior Target	90	35

⁎ Where length is measured from skull surface overlying entry ROI to target ROI

#### 2.3.2 Computer-assisted planning

**Fig. 2:**
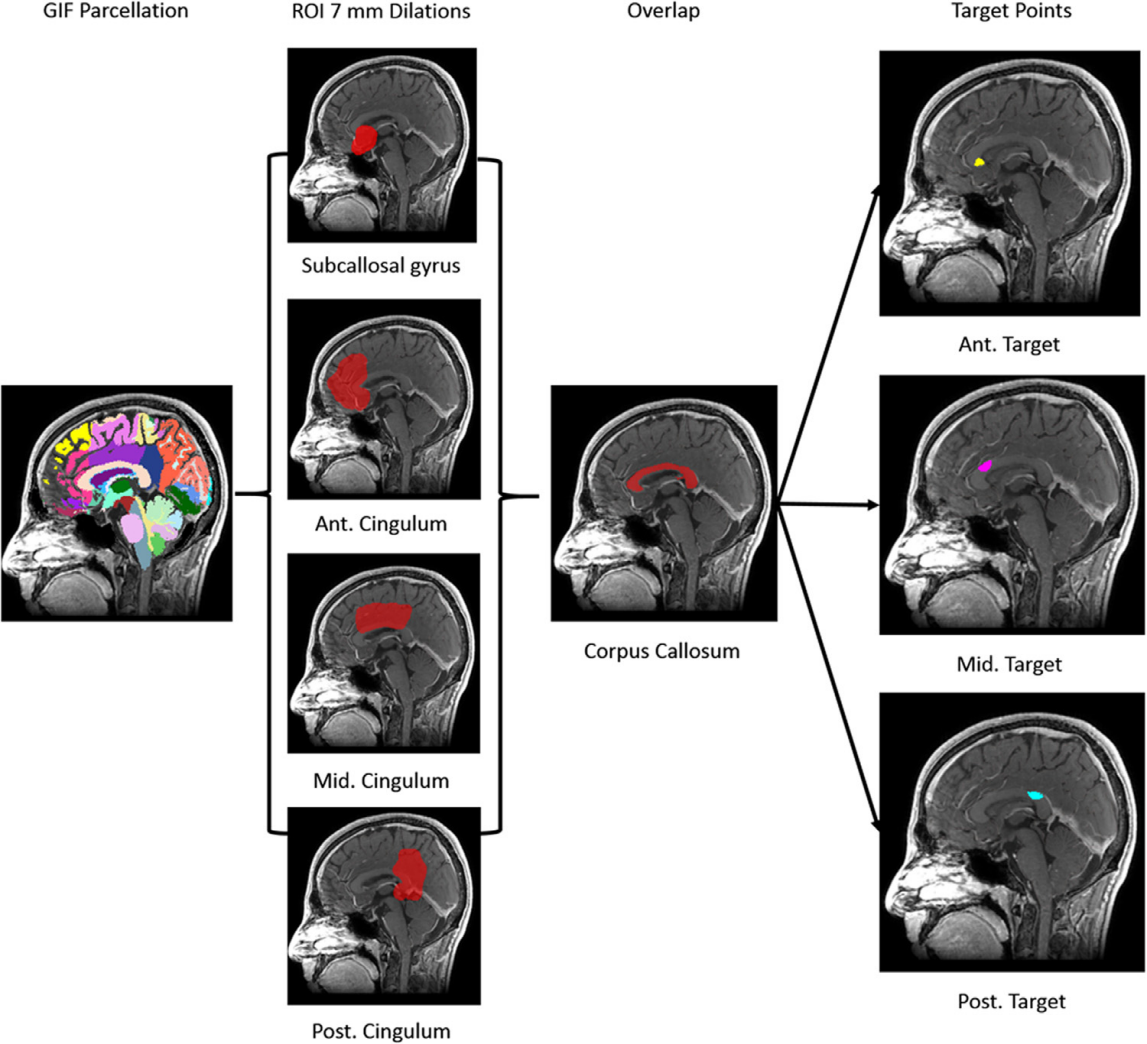
Legend: The patient-specific GIF parcellation was used to segment and dilate the subcallosal, anterior, middle and posterior cingulate gyri by 7 mm. The regions of overlap between the 7 mm dilated models e.g. overlap between subcallosal gyrus and anterior cingulum overlap, were then overlapped again with the corpus callosum resulting in 3 unique target points at the rostrum, genu and posterior body of the corpus callosum.

Computer-assisted planning was performed solely using the EpiNav™ platform (Sparks et al., 2017, 2016; Vakharia et al., 2017; Zombori et al., 2014) with trajectory entry and target points as well as default parameters determined from pilot data and expert consensus (see Table 1). EpiNav™ is an academic software developed at University College London, UK and Kings College London, UK that is available for research purposes without charge. To account for the wide spectrum of corpus callosum morphologies and to ensure parity with the manually planned trajectories the computer planning algorithm was set to undertake the ablation using 3 unique LITT trajectories. The trajectories consisted of: (1) a non-dominant frontal trajectory targeting the rostrum of the corpus callosum, (2) a non-dominant parietal trajectory targeting the genu of the corpus callosum and (3) a dominant frontal lobe trajectory targeting the posterior body of the corpus callosum. As is conventional for anterior two-third corpus callosotomy the splenium was spared in all cases. Based on surgeon preference the extent of ablation of the posterior body of the corpus callosum can be varied. Target points were generated in an automated fashion from the GIF parcellation through a series of morphometric dilations and Boolean operations (see Fig. 2). In brief, the callosum and subcallosal, anterior, middle and posterior cingulate cortices were segmented from the GIF parcellation. The subcallosal gyrus and anterior, middle and posterior cingulate cortices were then dilated by 7 mm and regions of overlap were then selected to create 3 regions of interest. The resulting regions were then constrained to their overlap with the corpus callosum to generate the anterior, middle and posterior target regions. Due to patient-specific anatomical and vascular variability, restricting trajectories to a single gyral entry region could lead to the algorithm finding a local optima and not the global optimum within the feasible search regions. To overcome this phenomenon 5 trajectories were initially planned and returned to the user as part of the automated algorithm in all patients. The user (in this case author VNV) then reviewed and selected the most feasible dominant frontal trajectory (between superior and middle frontal gyrus entry points) and non-dominant parietal trajectory (between superior parietal lobule and angular gyrus entry points) to be used in conjunction with the non-dominant frontal trajectory.

#### 2.3.3 Parameter optimisation

The algorithm optimises a number of different parameters that are considered by the neurosurgeon during trajectory planning. These include the intracerebral trajectory length, drilling angle to the skull (from the orthogonal), the proportion of the catheter within the corpus callosum, minimum distance from vasculature and size of the avascular corridor, expressed as a risk score. The risk score is a normalised score of the cumulative distance from vasculature for the trajectory at 128 nodes equally placed along the trajectory. A score of <1 signifies that the trajectory is >3 mm from vasculature along its entire course. For a detailed explanation of risk score and distance to vasculature calculation see (Sparks et al., 2017).

### 2.4 Vascular imaging acquisition

Digital subtraction angiography had been acquired as part of the routine care prior to performing SEEG at the study institution. This was performed through a femoral puncture in an interventional radiology suite equipped with biplane fluoroscopy. Late phase bilateral internal carotid artery injections were performed in all patients to ensure visualisation of both arteries and veins. A vessel extraction algorithm and thresholding were applied to the images. Accuracy of vessel segmentation and registrations were manually checked in all cases.

### 2.5 Manual planning

Manual planning was performed by two independent blinded external neurosurgeons (YB and JTW) with expertise in LITT for corpus callosotomy on all ten patients using the T1+Gad images. Entry and target points were manually selected by the neurosurgeon based on their current clinical practice. The neurosurgeons were blinded to the pre-calculated computer-assisted plans. In all 10 patients the two experts planned 3 trajectories per patient in keeping with their current clinical practice. Feasibility of the trajectories was based on the individual neurosurgeon performing the manual planning, or reviewing the computer-assisted plans, and was defined as feasible if the neurosurgeon was willing to implement the trajectory based on their current clinical practice.

### 2.6 Ablation volume generation

Simulated laser ablation cavities for both the computer-assisted and manually generated trajectories were generated based on the number of expected pull-backs of the catheter assuming a 5-15 mm ablation diameter, as expected using the Visualase™ system (Medtronic Inc., Minnesota, USA). The number of pull-backs was calculated by dividing the length of the laser catheter within the corpus callosum segmentation by 7 mm. The simulated ablation cavities relating to all three laser catheters were then saved as regions of interest and used as part of the tractography validation.

### 2.7 Probabilistic tractography

Diffusion-weighted MRI data were acquired using a single-shot EPI readout with 2 mm isotropic resolution (TE/TR = 74.1/7600 ms) and a total of 115 volumes were scanned using a multi-shell approach (11, 8, 32, and 64 gradient directions at b-values: 0, 300, 700, and 2500 s/mm^2^, respectively). A single b=0-image with reverse phase-encoding was also acquired for distortion correction. Diffusion data were corrected for scanner drift (Vos et al., 2017) and eddy current-induced distortions, subject movement and susceptibility-induced distortions using FSL v5.10 eddy and top-up tools (Andersson et al., 2003; Andersson and Sotiropoulos, 2016). Fibre orientation distributions were estimated in each voxel using multi-tissue constrained spherical deconvolution in MRtrix3 (Jeurissen et al., 2014; Smith et al., 2012).

**Fig. 3:**
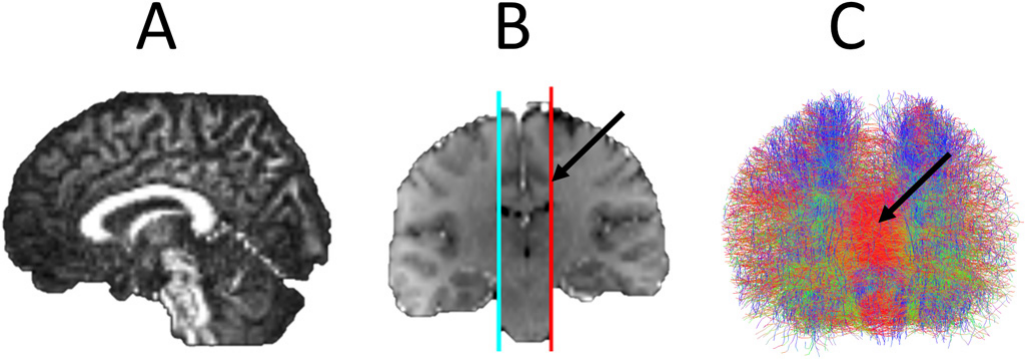
Legend: Probabilistic fibre tractography of interhemispheric connectivity. A) FA image in sagittal place revealing corpus callosum. B) Coronal T1 image registered to FA image with the seed (cyan) and inclusion (red) regions drawn manually on a paramedian sagittal plane at the level of the depth of the cingulate sulcus (arrow). These seed plane depicts the voxels from which streamlines are generated and the inclusion place depicts the voxels through which these streamlines must pass to be selected. C) Anterior view of resulting tractography consisting of 5000 streamlines with corpus callosum fibres visible in red (arrow).

**Fig. 4:**
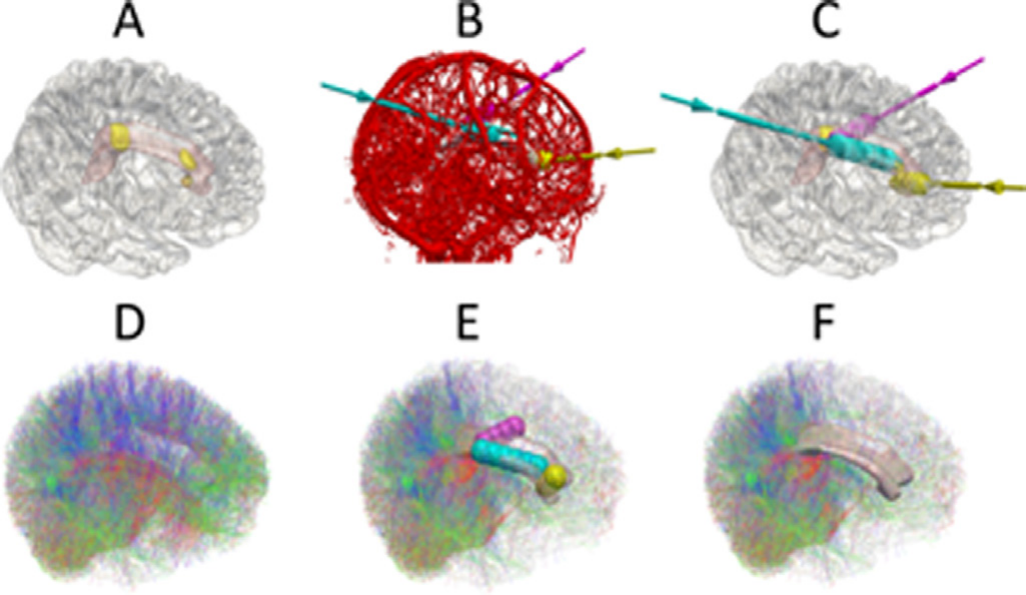
Legend: Trajectory planning and tractographic validation. Superior and lateral view of A) the right hemisphere (transparent white) with underlying corpus callosum (pink) and computer-assisted planning target points (yellow). B) Result of computer-assisted planning with three trajectories and vascular segmentation. C) Computer-assisted planning trajectories without vascular segmentation revealing the calculated number of ablations and pull-backs required for each trajectory. D) Tractography of right and left hemisphere connectivity. E) Simulated ablations for the trajectories and the corresponding effect on the hemispheric connectivity. F) The corresponding change in hemispheric connectivity shown without the simulated ablations revealing complete disconnection of streamlines passing through the corpus callosum.

Tractography of right and left hemispheric connectivity was performed using MRtrix3 employing constrained spherical deconvolution estimated fibre orientation distributions and streamline propagation by 2^nd^ order integration over fibre orientation distributions (see (Tournier and , F. Calamante, 2010) for a detailed description). Anatomically constrained tractography was applied to prevent biologically implausible streamline generation (Smith et al., 2012) using the tissue segmentation from the GIF parcellation. A seed region of interest was manually drawn on a paramedian sagittal plane at the depth of the cingulate sulcus on the T1 image registered to the diffusion image space. A corresponding symmetrical inclusion region of interest was drawn at the same location on the contralateral hemisphere. A total of 5000 streamlines were generated for each patient (See Fig. 3). Calculated ablation cavities from both the computer-assisted and manually generated trajectories were added as exclusion zones to the tractography in each patient to simulate the loss of right and left hemisphere connectivity following LITT corpus callosotomy (See Fig. 4). Residual hemispheric connectivity were defined as regions of the corpus callosum through which propagated streamlines remained after application of the exclusion masks derived from the simulated ablation cavities. These regions were binarised and converted to 3D meshes for volume measurement.

### 2.8 Statistical analysis

Comparison of trajectory metrics between computer-assisted and manually generated plans from two independent blinded expert neurosurgeons was performed by implementing an ANOVA model and appropriate post-hoc tests with Bonferroni correction. All statistical analyses were performed using SPSS25 (Armonk, NY: IBM Corp.).

## 3 Results

### 3.1 Safety metrics

Computer-assisted planning was able to calculate feasible trajectories in all 10 patients. Compared to both of the manually derived trajectory sets the computer-assisted plans returned a statistically significant reduction in the risk score (p < 0.001) and minimum distance to vasculature (mm) (p < 0.001) (see Table 2). There was no significant difference between the computer-assisted planning and the manually planned trajectories, or between the two sets of manually planned trajectories, for intracerebral length (p = 0.213) and drilling angle to the skull (p = 0.098).

**Table 2:** Comparison of trajectory metrics between expert manual and computer assisted plans.

Parameters	Manual Plan 1 *(Mean ± SD)*	Manual Plan 2 *(Mean ± SD)*	Computer-assisted plan *(Mean ± SD)*	p-value
Intracerebral length (mm)	93.6 ± 22.7	100 ± 26.6	89.7 ± 21.9	0.213
Drilling angle to skull (deg)	20.8 ± 10.0	23.9 ± 14.1	17.5 ± 9.3	0.098
Risk score	1.3 ± 0.1	1.3 ± 0.1	1.1 ± 0.1	0.000*
Minimum distance to vasculature (mm)	0.9 ± 1.0	0.8 ± 0.8	1.9 ± 1.2	0.000*

### 3.2 Tractography validation

**Fig. 5:**
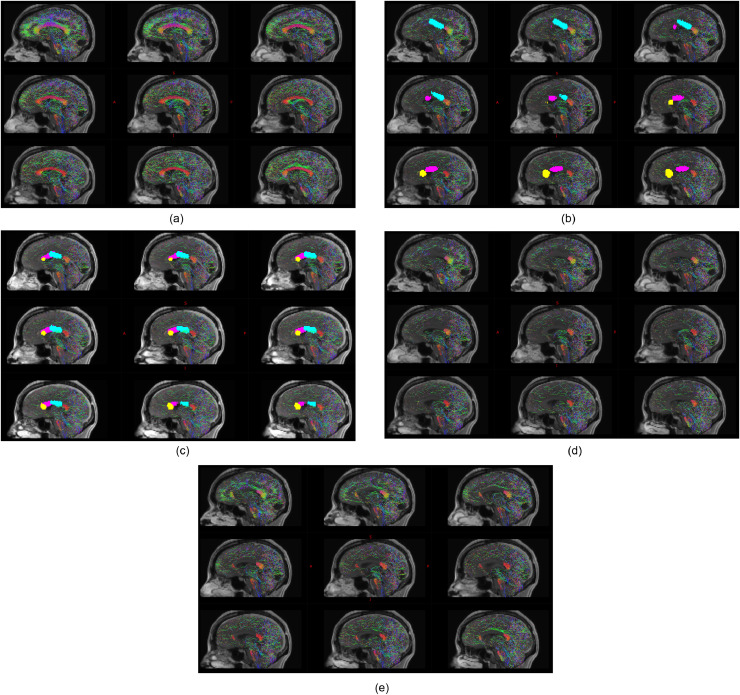
Legend: Example of residual interhemispheric connectivity identified from probabilistic tractography shown on median and paramedian sagittal planes. Streamline directionality encoded as red: right-left, blue: superior-inferior and green: anterior-posterior. A) Baseline interhemispheric probabilistic tractography in a single subject. B) Corresponding computer-assisted and C) manual plan derived estimated ablation volumes with the non-dominant frontal trajectory to the rostrum of corpus callosum shown in yellow, the non-dominant parietal trajectory to the genu of corpus callosum shown in magenta and the dominant frontal trajectory to the posterior body of the corpus callosum shown in cyan. D) Simulated effect of computer-assisted and E) manual plan estimated ablation cavities on structural interhemispheric connectivity, revealing residual connectivity at the genu of the corpus callosum associated with the manual plan compared to the computer-assisted plan (shown as red streamlines at the genu of the corpus callosum).

Based on the simulated ablation cavities, 4 out of 10 patients and 2 out of 10 patients following expert 1 and 2 manual trajectory planning, respectively, had residual hemispheric connectivity through the anterior two-thirds of the corpus callosum compared to 1 out of 10 following computer-assisted planning. In all cases, the residual interhemispheric connectivity was at the anterior-most aspect of the genu of the corpus callosum and related to cortical vasculature restricting the non-dominant frontal lobe trajectory from targeting the rostrum. The mean volume of the unablated anterior two-thirds corpus callosum was 0.69 cm^3^ compared to an intentionally unablated splenium volume of 3.5 cm^3^ and total corpus callosum volume of 15.4 cm^3^ (see Fig. 5).

## 4 Discussion

### 4.1 Key results

We show that computer-assisted planning was able to successfully plan feasible trajectories for LITT corpus callosotomy with a significantly improved risk score and minimum distance from vasculature. Employing probabilistic tractography of interhemispheric connectivity and simulated ablation cavities we demonstrate residual interhemispheric connectivity in 4/10 cases following expert 1 and 2/10 cases following expert 2 manual planning compared to 1/10 cases following computer-assisted planning.

### 4.2 Corpus callosotomy

Anatomically the corpus callosum lies within the interhemispheric fissure and is intimately related to the lateral ventricles. It is subdivided into the rostrum, genu, body and splenium. Open anterior two-thirds corpus callosotomy is performed through a bicoronal incision and midline craniotomy to expose the superior sagittal sinus. Interhemispheric dissection is performed with the callosomarginal and pericallosal arteries being identified and preserved (Smyth et al., 2017). Corpus callosotomy is then performed through microsuction and sharp dissection until the ependyma of the lateral ventricle or clefts of the septum pellucidum are encountered (Joseph et al., 2011). In most surgical practices, anterior two-thirds corpus callosotomy is performed initially with a second stage complete corpus callosotomy being performed if anterior disconnection fails to bring seizure control, to minimise the risk of developing a disconnection syndrome (Graham et al., 2016). For this reason, we have chosen to simulate anterior two-thirds callosotomy and not complete callosotomy. Other post-operative complications associated with corpus callosotomy are usually transient but may include language impairments, neuropsychological impairments, hemiparesis and urinary incontinence. The adverse effects associated with corpus callosotomy are usually less severe and better tolerated in children.

Descriptions of laser corpus callosotomy are limited to small case series, but initial results demonstrate that it is a feasible minimally invasive alternative to primary open anterior two-thirds corpus callosotomy or as a second stage completion callosotomy (see Table 3). Further studies are required to determine if laser corpus callosotomy could be used as a minimally invasive first line therapy.

**Table 3:** Summary of case reports in the literature

Authors	Number of patients	Number of trajectories	Objective	Outcome	Complications
Ho et al (2016)^8^	1	1	Second stage completion callosotomy following failed open anterior two-thirds corpus callosotomy	>50% seizure frequency reduction 4 months after ablation	Nil
Pruitt et al (2017)^9^	3	3	Not reported	Not reported	One patient suffered from catheter malposition
Lehner et al (2017)^7^	5	3	Anterior 2/3 corpus callosotomy	>80% seizure frequency reduction in 4 patients	One patient suffered a misplaced device requiring a second surgery. Another patient suffered a cortical haematoma at the entry site.
Tao et al (2018)^25^	2	2	Anterior 2/3 corpus callosotomy	Freedom from disabling seizures at 18 months in one patient and >90% seizure frequency reduction at 7 months	Nil
Palma et al (2018)^10^	3	Not reported	Second stage completion callosotomy following failed open anterior two-thirds corpus callosotomy	Not reported	Nil
Ball et al (2018)^26^	1	2	Anterior 2/3 corpus callosotomy	Engel 2 outcome	Nil
Karsy et al (2018)^27^	1	3	Anterior 2/3 corpus callosotomy	Not reported	Not reported

### 4.3 Computer-assisted planning and LITT corpus callosotomy

Computer-assisted planning has previously been described for stereotactic trajectory planning associated with deep brain stimulation (Essert et al., 2012), stereoelectroencephalography (De Momi et al., 2014; Sparks et al., 2017; Vakharia et al., 2018c), brain tumour biopsy (Marcus et al., 2019) and laser ablation of the mesial temporal lobe (Vakharia et al., 2018b) as a means of optimising and objectively standardizing clinical practice. This is the first description of an automated algorithm for optimising laser trajectories associated with anterior two-thirds corpus callosotomy. LITT is a novel minimally invasive method of creating thermal ablations within the brain that can be modulated in near real-time through MR thermography and application of the Arrhenius damage model (Lagman et al., 2017). A laser fibre and cooling catheter are stereotactically implanted into regions of the brain and ablations 5--20 mm in diameter are performed (Hoppe et al., 2017). The catheter can then be withdrawn and consecutive ablations performed to form a confluent ablation cavity. Based on a conservative estimate of a 15 mm ablation diameter we model 7 mm pull-backs for this purpose. The corpus callosum has a curved morphology whilst the laser catheters are currently restricted to linear trajectories. As a result, multiple trajectories are required to ensure that the corpus callosum is ablated to prevent residual connectivity and persistent seizure propagation (Ball et al., 2018; Karsy et al., 2018; Lehner et al., 2018; Tao et al., 2018). To attain this we aimed to automatically derive patient-specific target and entry points that would ensure interhemispheric disconnection through the corpus callosum whilst optimising the safety metrics considered by surgeons when planning stereotactic trajectories. There is currently no consensus within the literature regarding the use of 2 or 3 laser catheter trajectories. Two catheters may reduce the potential risk of implantation but could also result in incomplete disconnection. Based on expert experience we have therefore developed a 3 catheter algorithm to mirror their current clinical practice.This was achieved through the use of patient-specific whole brain parcellations in which morphometric dilations and Boolean operations were performed (see Fig. 2).

An important consideration during trajectory planning is haemorrhage risk. In clinical practice, we choose avascular corridors between the entry and target points to prevent conflict with intracerebral vessels. Here, we quantify the size of the avascular corridor using a risk score and also measure the minimum distance (mm) to vasculature. See (Sparks et al., 2017) for a formal description of the risk calculation. We make the pragmatic assumption that the greater the size of the avascular corridor the smaller the chance of haemorrhage. We accept that this has not been validated, as doing so would require a prohibitively large observational series, but we have incorporated this metric into the computer-assisted planning algorithm as it closely replicates established clinical practice. Another important consideration for maximising distance from vasculature is the heat-sink effect associated with blood flow in close proximity to the intended ablation cavity (Jermakowicz et al., 2018). Other factors that are optimised include the intracerebral length of the trajectory in order to prevent unnecessary parenchymal transgression, orthogonal drilling angle to the skull to prevent skiving during drilling and consequent inaccurate bolt/catheter placement and proportion of the trajectory within the corpus callosum.

Thermal injury to the adjacent fornix and cingulate gyri must also be avoided to prevent memory impairment and neuropsychological dysfunction, respectively. Given that the extent of the ablation cavity is modulated in near real time MR thermography we are unable to model this. Computer-assisted planning trajectories tended to have a more lateral and dorsal position within the corpus callosum compared to manually planned trajectories. The lateral positioning is due to the location of the pericallosal arteries lying on the dorsum of the corpus callosum in the midline. Injury to these could result in stroke or catastrophic haemorrhage. Additionally, the fornices lie closest to the posterior body of the corpus callosum in the midline. A potential consequence of a more lateral and dorsal trajectory, however, is increased cingulate cortex ablation. A prospective clinical trial would be required to compare the neuropsychological sequelae and safety of the trajectories.

Post-operatively, adequate disconnection following LITT corpus callosotomy has been described through a multimodal approach including contrast-enhanced MRI, diffusion-weighted imaging, functional MRI, cortico-cortical evoked potentials and resting EEG (Lehner et al., 2018). In addition, diffusion-weighted imaging and tractography alone have been shown to be highly accurate methods of quantifying post-callosotomy disconnection following open surgery (Choudhri, A.F., Whitehead, M.T., McGregor, 2013). As we are comparing simulated ablation cavities between computer-assisted and manual trajectory plans in the same patients we implemented probabilistic tractography to validate the extent of interhemispheric disconnection through the corpus callosum between the two methods. The different anatomical regions of the corpus callosum have been shown to demonstrate a consistent topographic organisation. The rostrum of the corpus callosum principally connects the orbitofrontal regions and the genu, also known as the forceps minor, connects the medial and lateral surfaces of the prefrontal cortex. The mid-body of the corpus callosum connects the pre-motor cortices whilst the posterior body connects the motor and sensory cortices. Posterior parietal, temporal and occipital connectivity pass through the anterior, middle and posterior aspects of the splenium respectively. We show that the extent of callosal disconnection through the rostrum, genu and body was no worse with computer-assisted planning compared to manually planned trajectories. In cases where residual connectivity was present after the application of simulated ablation cavities, this was on average 0.69 cm^3^. As we have not prospectively performed ablations in these patients, we are unable to comment on the clinical significance of this.

### 4.4 Limitations

The principal limitation of this feasibility study is that the LITT ablations are simulated and have not been prospectively implemented in patients. The corresponding cavities are modelled based on a 5-15 mm cylindrical ablation diameter (Jermakowicz et al., 2018; Vakharia et al., 2018b). Subsequently, we are unable to compare the corresponding outcomes with regards to alterations in seizure frequency or potential surgical morbidity were the trajectories implemented as part of LITT disconnection procedures. We feel this feasibility study is a necessary first step to ensure the computer-assisted planning algorithm is able to satisfy the requirements of anterior two-thirds of the corpus callosum under simulated conditions prior to undertaking a prospective clinical validation study. The simulated ablation volumes and number of catheter pull-backs required to achieve the desired ablation volume are based on a cylindrical ablation diameter. The Visualase™ product literature suggests that ablation diameters of between 5-20 mm may be achieved, but due to asymmetrical heat sinks, we conservatively model the maximum ablation diameter as 15 mm with 7 mm pullbacks to generate confluent ablation cavities. Heat dissipation to the adjacent white matter and cingulate cortices may also cause the surgeon to limit the ablation to prevent unintended damage to these structures. To date, no validated non-linear method exists to estimate the effect of heatsinks on thermal ablation cavities on a patient-specific basis, but this would improve the accuracy of the simulation.

Secondly, for the calculation of the risk score, we utilised vascular segmentations derived from DSA. We acknowledge that DSA is not routinely acquired for LITT procedures. The patients were selected from a prospectively maintained database of SEEG procedures and subsequently, DSA had already been acquired as part of their routine care. The algorithm would also work with MRV/A or CTA derived vascular segmentations. Given that the same segmentations were used for the calculation of the risk scores for both manual and computer-assisted plans this would not affect the comparison.

Finally, the automated entry and target point generation are dependent on the whole brain GIF parcellation. We have not tested the algorithm on other common whole brain parcellations, such as FreeSurfer (Martinos Centre for Biomedical Imaging, Charlestown, MA). Inaccuracies in the whole brain parcellation due to poor initial image quality must be checked prior to performing automated trajectory planning as this may adversely affect the trajectory planning.

## 5 Conclusion

Computer-assisted LITT anterior two-thirds corpus callosotomy trajectory planning appears feasible with a three trajectory technique. Compared to expert manually planned trajectories in the same patients, the computer-assisted planning algorithm was able to significantly optimise a number of heuristic parameters that surgeons employ during manual stereotactic trajectory planning. In addition, simulated ablation cavities and subsequent tractography revealed residual interhemispheric connectivity in 1 of 10 cases following computer-assisted planning compared to 2 and 4 of 10 cases following expert manual planning. Computer-assisted planning, therefore, provides a systematic and objective method of trajectory planning that may lead to standardised care. This has especially important implications for novel techniques, where initial learning curves are present at introduction. Prospective validation studies are now needed so that therapeutic efficacy can be determined. Future work is now focused on determining whether fewer catheter trajectories can be employed on an individual basis from the anatomical and morphological characteristics of the corpus callosum. More accurate modelling of heat dissipation in this region would also allow greater inferences regarding potential damage to nearby critical structures, such as the cingulate gyri and the fornix, as the algorithm is able to identify these as critical structures and minimise heat dissipation to them.

## Author contributions

VNV, RES and SBV were involved with study conception, technical expertise, acquisition and processing of the data. YB, JTW, ADM, CW and AS were involved with manual trajectory planning and/or providing clinical expertise. SO and JSD provided study supervision and critical revision of the manuscript. All authors reviewed the manuscript prior to submission.

## Funding Sources

NIHR UCLH/UCL Biomedical Research Centre, senior investigator schemes and Wellcome Trust (WT106882) / Wellcome/EPSRC (203145Z/16/Z).

## Declaration of Competing Interest

The authors have no conflicts of interest to declare.

## Appendix Supplementary materials

Image, image 1
