# Supplementary material for: Computer-assisted planning for minimally invasive anterior two-thirds laser corpus callosotomy: A feasibility study with probabilistic tractography validation
Source: Neuroimage Clin. 2020 Jan 13;25:102174. doi: 10.1016/j.nicl.2020.102174 (PMC6994706; doi:10.1016/j.nicl.2020.102174)
Supplement: Supplementary file 1 [file mmc1.docx]

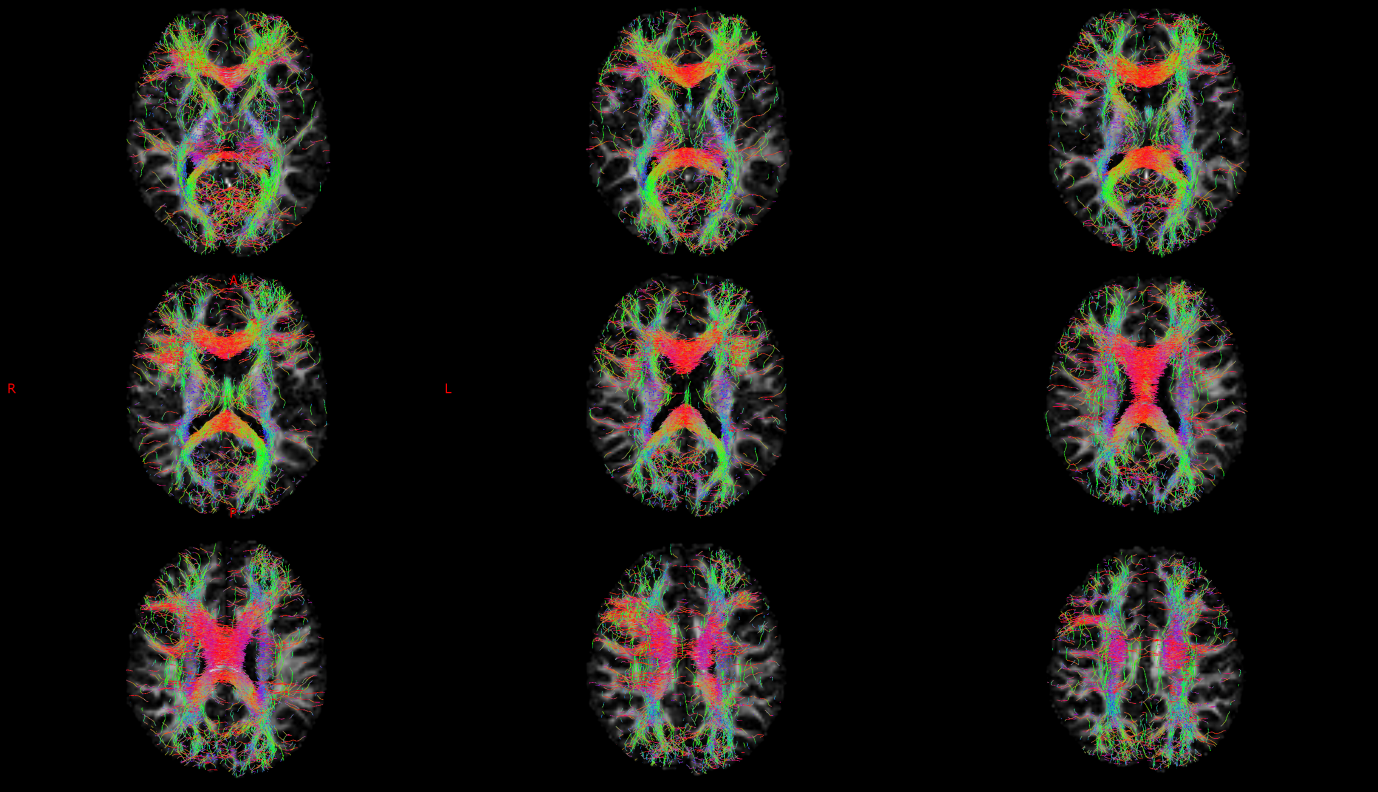


fig-1a


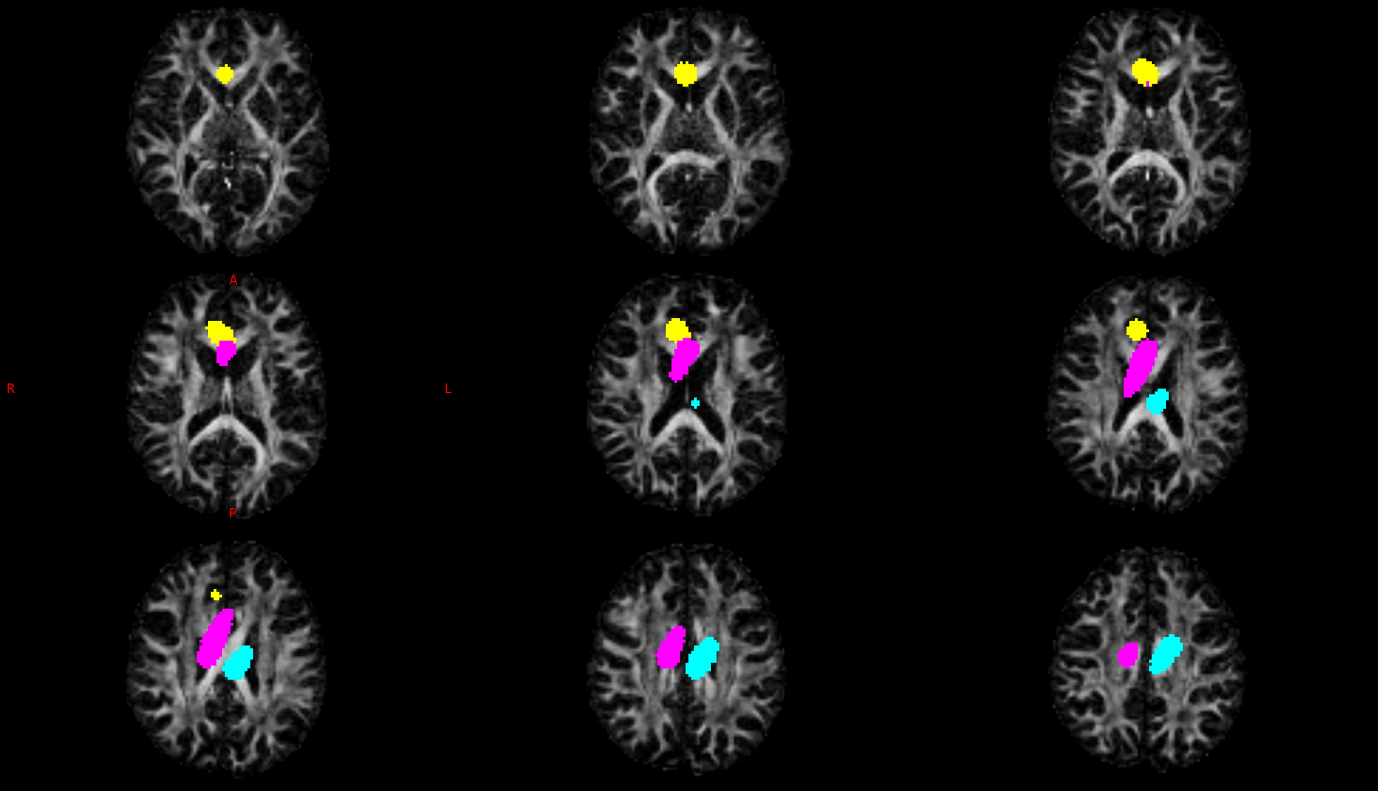


fig-1b


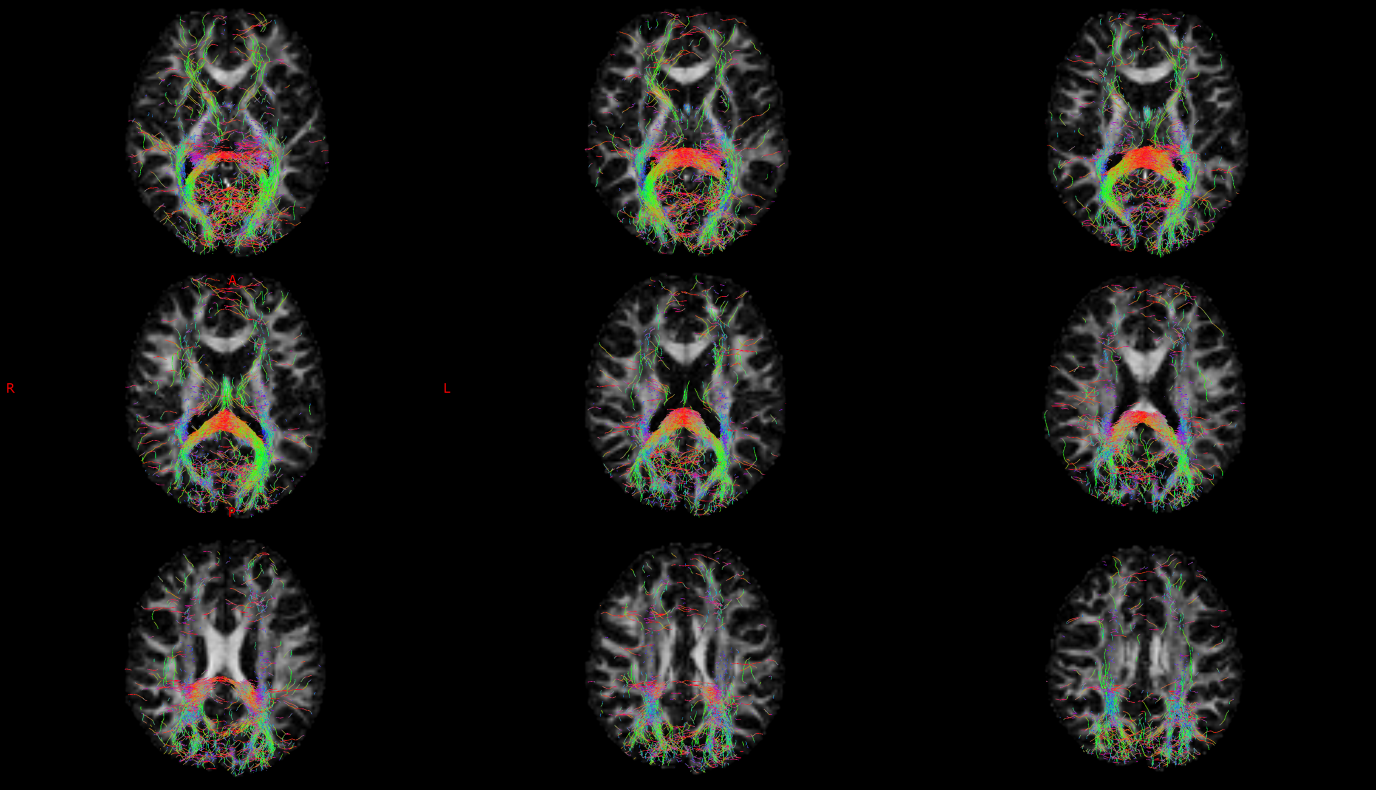


fig-1c

Supplementary Figure 1 Legend: Axial 3x3 lightbox images spanning the corpus callosum with A) interhemispheric probabilistic tractography streamlines overlaid on FA image, B) estimated ablation cavities associated with the three computer-assisted planning derived laser trajectories and C) simulated effect of ablation cavities on probabilistic tractography streamlines demonstrating complete interhemispheric disconnection through the anterior two-thirds of the corpus callosum and intact streamlines through the splenium (forceps major).
